# Identification of Regulatory Elements That Control PPARγ Expression in Adipocyte Progenitors

**DOI:** 10.1371/journal.pone.0072511

**Published:** 2013-08-29

**Authors:** Wen-Ling Chou, Andrea Galmozzi, David Partida, Kevin Kwan, Hui Yeung, Andrew I. Su, Enrique Saez

**Affiliations:** 1 Department of Chemical Physiology and The Skaggs Institute for Chemical Biology, The Scripps Research Institute, La Jolla, California, United States of America; 2 Department of Integrative Structural and Computational Biology, The Scripps Research Institute, La Jolla, California, United States of America; Georgia Regents University, United States of America

## Abstract

Adipose tissue renewal and obesity-driven expansion of fat cell number are dependent on proliferation and differentiation of adipose progenitors that reside in the vasculature that develops in coordination with adipose depots. The transcriptional events that regulate commitment of progenitors to the adipose lineage are poorly understood. Because expression of the nuclear receptor PPARγ defines the adipose lineage, isolation of elements that control PPARγ expression in adipose precursors may lead to discovery of transcriptional regulators of early adipocyte determination. Here, we describe the identification and validation in transgenic mice of 5 highly conserved non-coding sequences from the PPARγ locus that can drive expression of a reporter gene in a manner that recapitulates the tissue-specific pattern of PPARγ expression. Surprisingly, these 5 elements appear to control PPARγ expression in adipocyte precursors that are associated with the vasculature of adipose depots, but not in mature adipocytes. Characterization of these five PPARγ regulatory sequences may enable isolation of the transcription factors that bind these *cis* elements and provide insight into the molecular regulation of adipose tissue expansion in normal and pathological states.

## Introduction

Obesity is a risk factor in multiple diseases, including type 2 diabetes, cardiovascular disease, and cancer [1]. The emergence of obesity as a grave public health problem has focused interest on adipose tissue and fat cell function. Adipose tissue is an important metabolic and endocrine organ that is critical for energy balance and insulin sensitivity [2]. White adipose tissue (WAT) serves as a storage site for excess energy, while brown adipose tissue (BAT) dissipates energy to generate heat. Adipocytes also secrete adipokines (e.g., leptin, adiponectin) that regulate multiple physiologic processes, including appetite and glucose homeostasis [3], [4]. In obesity, the ability of adipocytes to store lipids, dispose of glucose, and secrete adipokines is compromised. Obesity-driven adipocyte dysfunction is intimately linked to the development of systemic insulin resistance and type 2 diabetes [5], [6]. In response to a chronic energy imbalance, the number and the size of adipocytes increases to retain excess energy. Eventually, adipose tissue expansion is not sufficient to store surplus fatty acids and adipocyte-released lipids deposit in tissues such as liver and muscle where they dampen insulin action. A better understanding of how adipose tissue develops and expands is thus critical to devise new avenues to treat obesity and its associated complications.

Adipose tissue mass can expand throughout life [7]. Under normal circumstances, approximately 10% of human adipocytes are renewed each year [8]. Obesity can increase the rate of adipocyte proliferation and differentiation [9]. Because mature adipocytes are non-dividing, renewing or increasing the number of fat cells relies on the differentiation of proliferating adipose progenitors that are found in the stromal-vascular fraction of adipose depots [10]. Environmental stimulation (e.g., chronic high-fat feeding) induces adipose stem cells in this niche to commit to the preadipocyte lineage, which can then give rise to terminally differentiated adipocytes. While recent studies have identified cell-surface markers that allow isolation of progenitor cells with adipogenic potential [11]–[13], and lineage tracing analyses have shown that adipogenic precursors reside in the mural cell compartment of the adipose vasculature [13]–[16], little is known about the transcriptional events that prompt adipose progenitors to commit to the preadipocyte lineage (determination). Recent work has associated the zinc-finger protein Zfp423, its paralog Zfp521, and the factors Zfp467, Tcf7L1, and Ebf1 with preadipocyte determination, but the transcriptional regulation of early adipose commitment remains poorly understood [17]–[21].

In contrast, the major components of the transcriptional cascade that brings about preadipocyte to adipocyte terminal differentiation have been identified [22], [23]. PPARγ, a lipid-regulated transcription factor of the nuclear receptor family, is the master regulator of adipocyte terminal differentiation. Expression of PPARγ is required for fat cell formation [24]–[26]. Although PPARγ expression was thought to be associated primarily with differentiated adipocytes, a recent lineage tracing analysis using PPARγ-reporter strains has revealed the existence of immature PPARγ-expressing cells that reside in the adipose vasculature [14]. This population of PPARγ-expressing proliferating cells gives rise to the vast majority of adipocytes in the mature fat pad.

Because PPARγ expression is the defining feature of the adipose lineage, greater understanding of the transcription factors that control PPARγ expression in adipose progenitors may shed insight into the dynamics of adipose tissue expansion in normal and pathological states. In contrast to the attention that has been paid to pharmacologic activation of PPARγ, much less is known about the regulation of PPARγ expression, particularly during the early stages of adipose commitment. As a first step to discern the transcription factors that control the initial phases of adipocyte determination, we have carried out a comparative genomic analysis to identify conserved sequence elements in the 5′-flanking region of the PPARγ locus that may be responsible for its pattern of expression. We have isolated five elements that appear to be sufficient to recapitulate the tissue-specific pattern of PPARγ expression *in vivo*. These 5 non-coding DNA sequences from the 5′-flanking region of the PPARγ locus can drive expression of a reporter in adipose progenitors localized in the vasculature of white and brown fat pads. Interestingly, the ability of these sequences to activate transcription decreases as adipocyte differentiation proceeds. These findings indicate that these 5 *cis* elements behave as enhancers that control PPARγ expression at the earliest stages of adipocyte determination, but not during terminal differentiation.

## Results and Discussion

### Isolation of Conserved Genomic Regions that Regulate PPARγ Expression

**Figure 1:**
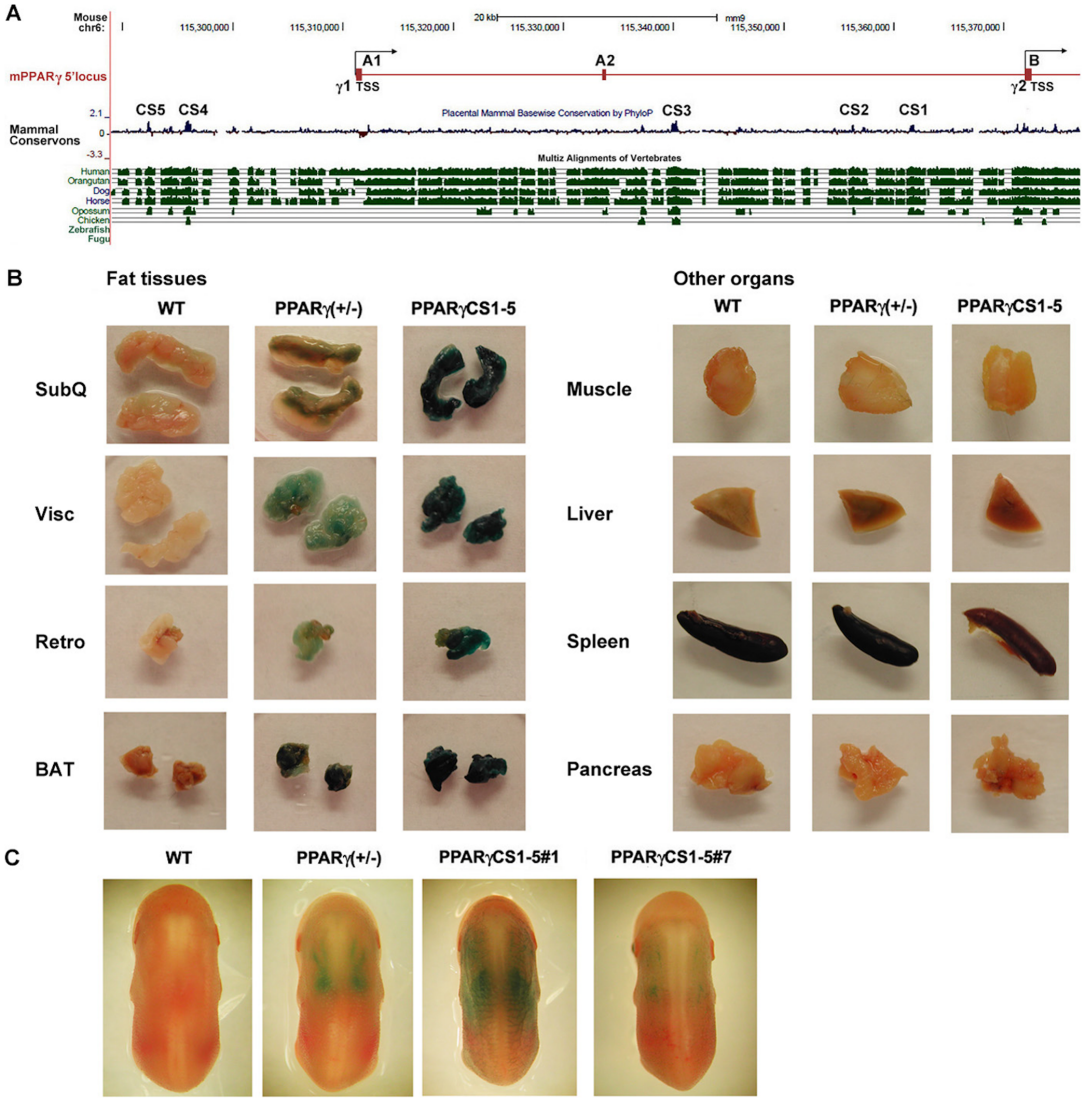
Identification and functional validation of genomic regions that regulate PPARγ expression *in vivo*. (**A**) Comparative analysis of 100 Kb of sequence upstream of the PPARγ2 transcriptional start site reveals 5 elements that are highly conserved across multiple mammalian species (indicated as CS1 to 5 in the UCSC genome browser schematic). (**B**) X-gal staining of subcutaneous (SubQ), visceral (Visc), and retroperitoneal (Retro) WAT, brown adipose tissue (BAT), and other organs from wild type, PPARγ (+/−), and PPARγ CS1-5_*LacZ* line 1 transgenic mice (6 weeks). Note that CS1 to 5 drive reporter expression in a similar tissue-specific pattern to that of *LacZ* expressed from the endogenous PPARγ locus. (**C**) X-gal staining of wild type, PPARγ (+/−), and PPARγ CS1-5_*LacZ* line 1 and 7 embryos at E14.5.

The tissue-specific pattern of expression of genes is thought to be primarily due to the action of enhancers, non-coding DNA sequences that are often located far away from the basal promoter of the gene whose transcription they control [27]–[29]. Comparison of sequence conservation across species can be useful to identify non-coding DNA sequences that behave as functional enhancers *in vivo* [30]. There are two major isoforms transcribed from the PPARγ locus, PPARγ1 and γ2 [31]. Each isoform is transcribed from a different promoter and alternative exon usage gives rise to two proteins that differ in the N-terminus. The PPARγ2 mRNA is expressed almost exclusively in adipose depots, while PPARγ1 exhibits a broader pattern of expression. Since our intention was to identify genetic elements that regulate PPARγ expression at the earliest stages of adipogenesis, we carried out a comparative genomic analysis of a 100 Kb genomic region upstream of the PPARγ2 transcriptional start site (TSS) that includes the PPARγ1 promoter. Five evolutionarily conserved sequences (CS1 to CS5), representing putative regulatory elements, were identified based on alignment of 30 mammalian species using the MULTIZ algorithm (Fig. 1A). These elements range in size from 357 to 991 bp and are >80% identical across mammals, similar conservation to that of PPARγ exons, suggesting that they could contain the regulatory sequences that control PPARγ expression. CS1, CS2, and CS3 are located between exon A2 and exon B (−11 to −32 Kb from the PPARγ2 TSS), while CS4 and CS5 are located upstream of the PPARγ1 exon A1 and far from the PPARγ2 TSS (∼−79 Kb) (exact genomic locations shown in Supplemental Table 1).

**Figure 2:**
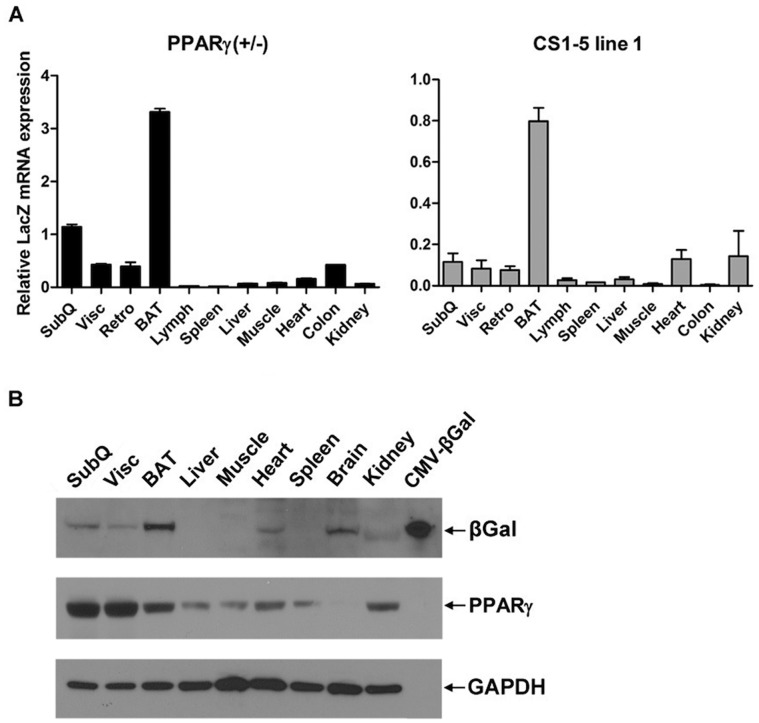
*LacZ* expression controlled by PPARγ conserved sequences 1-5 reflects the tissue-specific pattern of endogenous PPARγ expression. (**A**) Tissue distribution of *LacZ* mRNA expression in PPARγ (+/−) and PPARγ CS1-5_*LacZ* line 1 transgenic mice (5 weeks, n = 3), evaluated by RT-qPCR. Error bars denote mean ± S.D. (**B**) Western blot analysis of β-galactosidase and PPARγ levels in tissues of PPARγ CS1-5_*LacZ* line 1 mice (7 weeks, n = 2). An extract of HEK-293T cells expressing exogenous β-galactosidase served as positive control.

To evaluate the extent to which these sequences control PPARγ expression *in vivo* (i.e. behave as enhancer elements that dictate tissue-specific PPARγ expression), we cloned all 5 elements together into an Hsp68-*LacZ* reporter vector to generate PPARγ CS1-5_Hsp68-*LacZ* transgenic mice (referred hereafter as PPARγ CS1-5_*LacZ*; Supplemental Fig. 1). The Hsp68 minimal promoter was chosen because this is a widely used basal promoter for *in vivo* enhancer analysis [30]. To establish if these 5 conserved elements are sufficient to drive expression of the *LacZ* reporter in a pattern similar to that of endogenous PPARγ, we analyzed *LacZ* expression by X-gal staining in tissues of 5 independently-derived PPARγ CS1-5_*LacZ* transgenic lines. One line (line 1) showed very strong X-gal staining in brown fat and in all white adipose depots (Fig. 1B). To check the specificity of reporter expression, we analyzed *LacZ* expression in skeletal muscle, liver, spleen, and pancreas and found no X-gal staining in these organs (Fig. 1B and Supplemental Fig. 5). The pattern of X-gal staining in this PPARγ CS1-5_*LacZ* transgenic line mirrored that seen in PPARγ (+/−) heterozygous null mice in which an allele of PPARγ was targeted by an in-frame insertion of a neomycin-*LacZ* construct (β-geo) into exon 2 of PPARγ [24]. Analysis of *LacZ* expression across tissues by RT-qPCR and Western Blot indicated that the PPARγ CS1-5_*LacZ* transgene was expressed in a similar pattern to that of endogenous PPARγ (Fig. 2), with greatest expression of mRNA and protein in fat depots, and lower levels in selected other organs. This adipose-enriched pattern of expression of the transgene suggested that these 5 conserved sequences contain most of the regulatory elements necessary for tissue-specific PPARγ expression. Two additional PPARγ CS1-5_*LacZ* transgenic lines (lines 6 and 7) showed an identical, but weaker, pattern of X-gal staining and *LacZ* mRNA expression, indicating that the pattern of transgene expression we observed is not the consequence of integration effects.

During mouse development, PPARγ expression correlates with the appearance of the interscapular brown fat depot at embryonic day 14.5 (E14.5), and with the emergence of adipose progenitor cells that can be detected at postnatal day 1 and are associated with the vasculature of what becomes the white adipose tissue depots [14],[24]. To examine the extent to which the 5 conserved PPARγ sequences regulate PPARγ expression during development, we evaluated expression of the PPARγ CS1-5_*LacZ* transgene at E14.5 (Fig. 1C). X-gal staining in control PPARγ (+/−) embryos showed that, as reported, PPARγ expression at this stage is only evident in the brown fat depot. Line 7 PPARγ CS1-5_*LacZ* transgenic embryos showed weak, but clearly detectable X-gal staining that was spatially restricted to the location of the BAT depot. Line 1 transgenic embryos showed a strong pattern of X-gal staining that encompassed the BAT depot, but broadened beyond the staining pattern in control PPARγ (+/−) embryos. In this line, the one with highest transgene expression, the X-gal stain was additionally associated with what appeared to be the vascular network that underlies the epidermis, perhaps an indication that the transgene is active in cells that could form the basis of the subcutaneous fat layer that supports the dermis (Fig. 1C and Supplemental Fig. S2). It is probable that this additional X-gal stain is not detected in PPARγ (+/−) embryos because these embryos express only one copy of the *LacZ* reporter, while line 1 embryos are likely to have multiple copies of the reporter transgene, as is often the case in transgenic lines. Together with our results in adult tissues, these data indicate that the 5 conserved sequences we have identified play an important role in the tissue-specific regulation of PPARγ expression *in vivo*.

### Transcription Driven by Conserved PPARγ Sequences 1 to 5 Decreases during Adipocyte Differentiation

**Figure 3:**
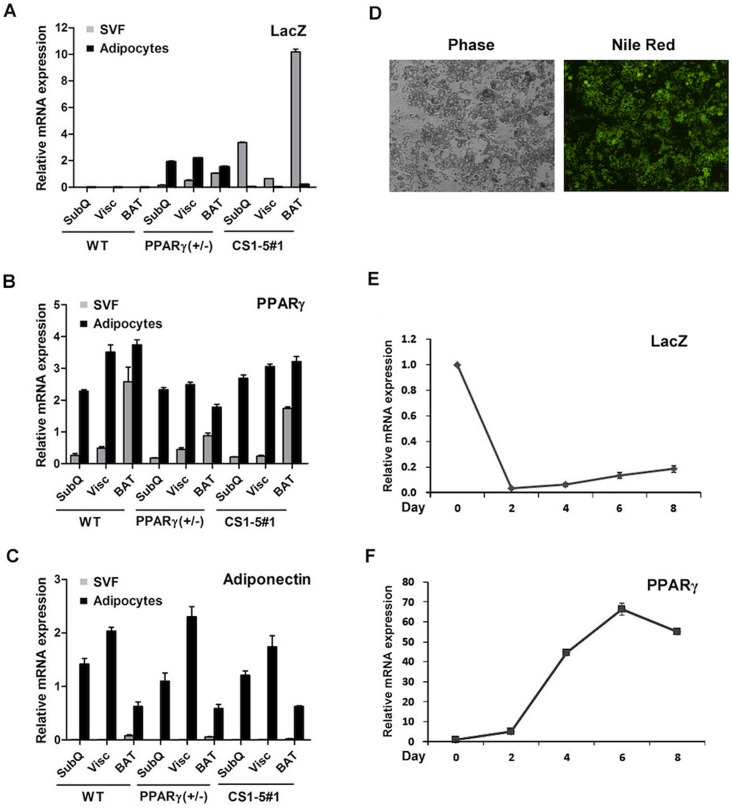
Conserved PPARγ sequences 1 to 5 are transcriptionally active in adipocyte precursors, but not in mature fat cells. Real-time qPCR analysis of *LacZ* (**A**), PPARγ (**B**), and adiponectin (**C**) expression in the stroma-vascular (SVF) and adipocyte fractions of fat pads derived from wild type, PPARγ (+/−), and PPARγ CS1-5_*LacZ* line 1 mice (6–7 weeks, n = 3 per group). Error bars denote mean ± S.D. (**D**) Conditionally immortalized SV cells from subcutaneous WAT of line 1 transgenic mice at day 8 post-induction of adipocyte differentiation. Nile-red stains adipocyte neutral lipids. Levels of *LacZ* (**E**) and PPARγ (**F**) mRNA expression during the course of adipocyte differentiation in these cells. Error bars denote mean ± S.D.

Adipocytes develop in coordination with the vasculature, which supplies oxygen, nutrients, and endocrine factors, and provides a niche for pericyte-derived adipocyte progenitors [16], [32]. To explore the compartment(s) within adipose depots where CS1 to 5 PPARγ sequences are transcriptionally active, we measured *LacZ* and PPARγ mRNA expression after separation of the stromal-vascular (SV) and adipocyte fractions of WAT and BAT depots of wild type, PPARγ (+/−), and transgenic PPARγ CS1-5_ *LacZ* mice. As expected, *LacZ* expression driven by the entire endogenous PPARγ locus (as in PPARγ [+/−] mice) was predominantly associated with the differentiated adipocyte compartment, particularly in WAT depots (Fig. 3A). In contrast, we found that the CS1-5 PPARγ sequences activated *LacZ* mRNA expression almost exclusively in the SV compartment, and not in the adipocyte fraction of either WAT or BAT. This pattern of *LacZ* expression observed in transgenic line 1 was confirmed in two other transgenic lines (lines 6 and 7; see Supplementary Fig. 3 for line 7 data). Endogenous PPARγ mRNA was detected in the SV fraction, but was significantly enriched in the adipocyte compartment, with no differences among mice of different genotypes (Fig. 3B). The quality of our fractions was verified by measuring expression of adiponectin, a mature adipocyte marker that could only be detected in the adipocyte fraction (Fig. 3C). These results indicate that PPARγ CS 1 to 5 are transcriptionally active only in the SV fraction that contains adipocyte progenitors and preadipocytes, as well as other cells that do not contribute to the adipose lineage. Interestingly, expression of transgenic *LacZ*, but not that derived from the endogenous locus (PPARγ [+/−] mice), was consistently higher in BAT compared to WAT (Fig. 3A), perhaps a reflection of the larger vascular network that is present in BAT.

To evaluate in detail the behavior of the PPARγ CS1-5_*LacZ* transgene during the course of adipocyte differentiation, we conditionally immortalized SV cells isolated from the subcutaneous white (inguinal) and brown fat depots of transgenic mice and measured *LacZ* expression at various time points after induction of differentiation. Cells derived from transgenic animals differentiated into adipocytes with normal frequency (Fig. 3D and Supplemental Fig. S4). Intriguingly, *LacZ* mRNA expression in cells isolated from transgenic mice decreased dramatically upon the induction of adipocyte differentiation and remained low in maturing adipocytes (Fig. 3E). In contrast, PPARγ expression was highly induced during differentiation (Fig. 3F). The opposing pattern of CS1-5-driven *LacZ* expression relative to that of endogenous PPARγ, and its association with the SV fraction rather than with the adipocyte compartment, indicated that these sequences could be responsible primarily for expression of PPARγ in the progenitors that give rise to the adipocyte lineage.

### PPARγ Conserved Sequences 1 to 5 are Transcriptionally Active in Adipose Precursors that Line the Vasculature of White and Brown Adipose Tissue

**Figure 4:**
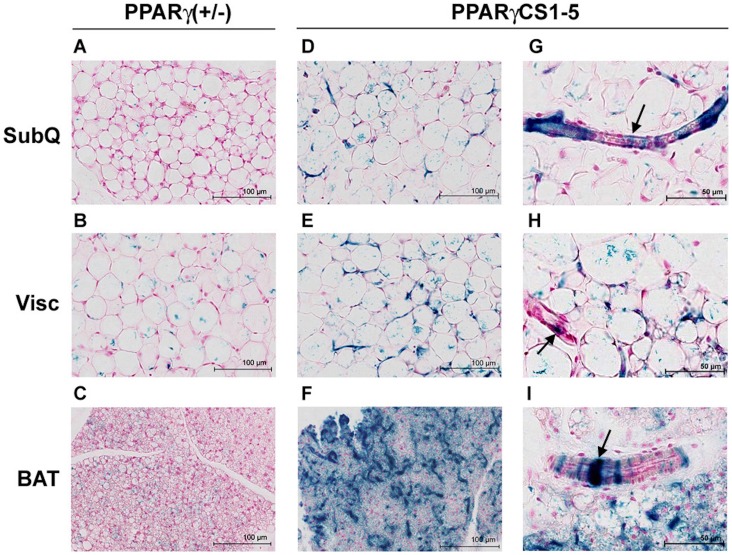
The PPARγ CS1-5 cassette is transcriptionally active in white and brown fat cell progenitors. Paraffin-embedded sections of X-gal stained subcutaneous (**A,D,G**) and visceral (**B**,**E**,**H**) WAT, and BAT (*C,F,I*) from PPARγ (+/−),and PPARγ CS1-5_*LacZ* line 1 transgenic mice (6 weeks). Note the perivascular nature of many *LacZ* expressing cells in transgenic fat pads (arrows), and the strong blue stain in much of the vasculature of transgenic BAT (**F**). Genotypes indicated on top.

**Figure 5:**
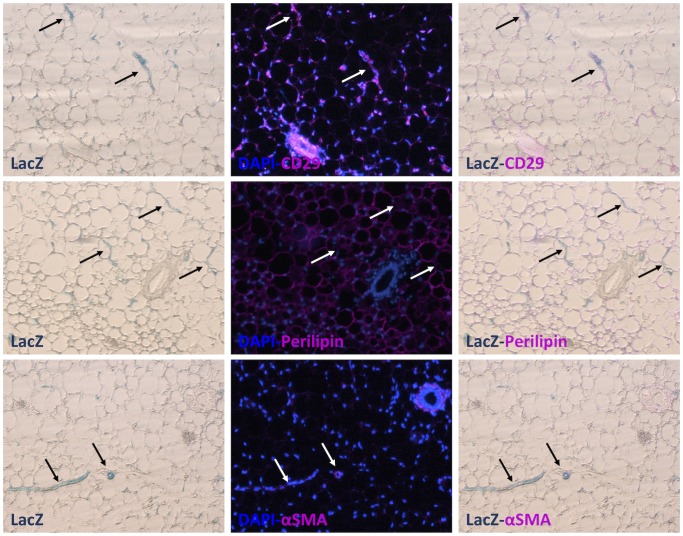
PPARγ CS1-5_*LacZ* positive cells express markers of adipose progenitors. Paraffin-embedded serial sections of X-gal stained subcutaneous WAT derived from PPARγ CS1-5_*LacZ* line 1 transgenic mice were analyzed by immunohistochemistry. Note that *LacZ* positive cells in transgenic fat pads express mural/endothelial/adipose progenitor cell markers (CD29, SMA), but not perilipin (mature adipocytes). Arrows point to several examples of the same *LacZ* positive cells in all serial sections, so that the overlap of markers can be evaluated.

Lineage tracing studies have taken advantage of the high stability of β-galactosidase protein to show that PPARγ is expressed in proliferating cells that reside in the adipose vasculature and give rise to mature adipocytes [14]. To explore the possibility that the CS1-5 elements could be responsible for PPARγ expression in adipocyte progenitors, we examined sections of X-gal stained WAT and BAT depots from PPARγ CS1-5_*LacZ* and PPARγ (+/−) mice. In PPARγ (+/−) fat pads, the X-gal stain was associated with mature adipocytes in all depots (Fig. 4A–C), with a few *LacZ* positive cells along some capillaries. In contrast, in PPARγ CS1-5_ *LacZ* transgenic fat pads the X-gal stain was detected in some mature adipocytes, but it was significantly more prominent along the vasculature of both WAT and BAT fat pads (Fig. 4D–F). The staining was particularly strong in sections of transgenic interscapular BAT, where the stain outlined many of the vessels present in this tissue (Fig. 4F). *LacZ* staining was present, not only in small capillaries, but also in a perivascular pattern in some larger size vessels. Images taken at higher magnification (Fig. 4G–I) revealed the presence of *LacZ* positive cells in the mural cell compartment of the vasculature, where adipocyte progenitors reside. No *LacZ* positive cells were detected, in association with the vasculature or otherwise, in X-gal stained sections of other tissues such as liver, skeletal muscle, and spleen (Supplemental Fig. 5). Immunohistochemical analysis of X-gal stained PPARγ CS1-5_ *LacZ* transgenic adipose tissue revealed that *LacZ* expression overlapped primarily with that of mural/endothelial/adipose progenitor cell markers (e.g., CD29 [10], [11], Smooth Muscle Actin [14]), but not with perilipin, a marker of mature fat cells (Fig. 5). These cytochemical and X-gal staining patterns, together with our data showing that conserved PPARγ sequences are active in the SV fraction but inactive in mature adipocytes, indicate that these elements play an important role in controlling PPARγ expression in adipocyte precursors, but not in mature fat cells.

Here, we have shown that 5 small non-coding sequences from the 5′-flanking region of the PPARγ locus are sufficient to activate PPARγ expression in adipocyte precursors of both, the white and the brown lineage. This is the first description of a transcriptional cassette that can direct gene expression to adipocyte progenitors. The extent to which all five elements are required to control PPARγ expression *in vivo* is presently under investigation. Interestingly, these five elements do not appear to play a role in the dramatic induction of PPARγ expression that accompanies adipocyte terminal differentiation. Members of the C/EBP family are thought to stimulate and sustain PPARγ expression at this later stage [22], [33]. Further characterization of these five PPARγ regulatory sequences may enable isolation of the *trans*-acting factors that bind these *cis* elements. Identification of the transcription factors that induce PPARγ expression through these elements in adipose progenitors will provide insight into the molecular regulation of normal adipose tissue turnover, its expansion in obesity, and perhaps its absence in lipodystrophies that remain to be associated with a molecular determinant.

## Methods

### Ethics Statement

Animal experiments in this work were limited to the harvest of tissues from humanely euthanized animals. The number of animals used was kept to the minimum necessary to insure data quality. The Scripps Research Institute’s Institutional Animal Care and Use Committee approved all procedures.

### Generation of Transgenic Mice

Conserved non-coding sequence elements from the PPARγ locus were cloned by PCR. A fragment containing all 5 conserved elements (CS1-5) in the endogenous orientation was cloned into the Hsp68-*LacZ* vector [30] to generate the PPARγ CS1-5_Hsp68-*LacZ* reporter. Transgenic mice were generated by pronuclear microinjection into C57BL/6 single cell embryos. Founders were identified by PCR (*LacZ* primers: *LacZ*-F: 5′-TTTCCATGTTGCCACTCGC-3′; *LacZ*-R: 5′-AACGGCTTGCCGTTCAGCA-3′) and bred to C57BL/6 mice to establish lines. F1 transgenics 5 to 7 weeks old were used for analysis unless otherwise indicated. All procedures were approved by the TSRI IACUC.

### X-gal Staining

Tissues were fixed in 1X PBS containing 2% formaldehyde, 0.2% glutaraldehyde for 30 min and rinsed three times 15 min each with wash buffer (2 mM MgCl_2_, 0.02% NP-40, 0.1% sodium phosphate, pH 7.3). They were then incubated with X-gal staining solution (1X PBS, 1 mg/mL X-gal, 2 mM MgCl_2_, 0.02% NP-40, 5 mM potassium ferrocyanide, 5 mM potassium ferricyanide) overnight at room temperature. Next day, tissues were washed in PBS, 70% ethanol, PBS, and photographed. Embryos (E14.5) were fixed (0.2% glutaraldehyde, 5 mM EGTA, and 2 mM MgCl_2_, 0.1 M sodium phosphate, pH 7.3) for 15 min and rinsed with wash buffer (2 mM MgCl_2_, 0.01% sodium deoxycholate, 0.02% NP-40, 0.1 M sodium phosphate, pH 7.3) for 15 min three times. Embryos were stained in X-gal solution for 1–3 hr at 37°C.

### Tissue Fractionation

Minced WAT and BAT depots were digested in isolation buffer (123 mM NaCl, 5 mM KCl, 1.3 mM CaCl_2_, 5 mM glucose, 0.1 M HEPES, pH 7.4, 4% BSA) containing 1.5 mg/mL collagenase A at 37°C for 1 hr. Digested tissues were passed through a 100 µm mesh, and the flow-through separated into SV and adipocyte fractions by centrifugation.

### Gene Expression and Protein Analysis

RNA was isolated using the NucleoSpin 96 RNA kit (Macherey-Nagel). Taqman-based real-time qPCR was performed using the Superscript III One-Step RT-PCR mix (Life Technologies). Multiplexed reactions (target and control) were run and target gene expression was normalized to the levels of 36B4. PPARγ and adiponectin primers/probes were obtained from ABI. *LacZ* probe 5′-6-FAM/CGGGTAAAC/ZEN/TGGCTCGGA TTA GGG/3IABkFQ -3′, *LacZ* primerF 350 5′-TCGGGATAGTTT TCTTGCGG-3′, and *LacZ* primerR 496 5′-TGGTAG TGGTCAAATGGCG-3′. For protein analysis, cells were lysed in RIPA buffer (100 mM Tris-HCl pH 7.4, 150 mM NaCl, 0.5% NP-40, 0.2% deoxycholate, 0.1% SDS, 1 mM EDTA, 0.5 mM DTT, and protease inhibitors). Tissues (harvested from mice perfused with PBS) were lysed in buffer with 50 mM Tris-HCl pH 7.4, 1 mM EDTA, 1 mM EGTA, 200 mM NaCl, 1% SDS, 1 mM DTT, and protease inhibitors. Antibodies: SV40 T Ag (Santa Cruz, Pab 108); PPARγ (Santa Cruz, E-8); β-Gal (Abcam, ab616); β-actin (Cell Signaling); GAPDH (Millipore).

### Immunohistochemistry

Paraffin sections of X-gal stained subcutaneous WAT (10 µm) were deparaffinized and rehydrated, permeabilized with PBS-Triton X–100 0.5% for 15 min and antigen retrieval was performed with PBS-SDS 1% solution for 10 min at room temperature. Blocking was performed in 10% FBS-PBS/Triton X–100 0.1% for 1 hr at room temperature. Anti-Integrin β1 (CD29, BD Pharmingen #558741), anti-Perilipin (Cell Signaling #9349) or anti-Smooth Muscle Actin (SMA, Dako #M0851) antibodies were applied (1∶200) overnight at 4°C in 5% FBS-PBS/Triton X–100 0.1% followed by 3 washes in PBS-Triton X–100 0.1%. Incubation with secondary antibodies (1∶500, AlexaFluor 488 donkey anti-rat IgG #A-21208, AlexaFluor 546 Donkey anti-rabbit IgG #A11035, AlexaFluor 546 donkey anti-mouse IgG #A10036) was performed in 5% FBS-PBS/Triton X–100 0.1% at room temperature for 1 hr; sections were then washed 3 times in PBS-Triton X–100 0.1%. Cell nuclei were stained with DAPI solution for 10 min at room temperature. Sections were then washed and mounted. Images were taken at 4X magnification and processed with Adobe Photoshop and ImageJ software.

## Acknowledgments

We thank Dr. R.M. Evans for providing PPARγ (+/−) mice, Drs. L. Pennacchio and E.M. Rubin for the Hsp68-*LacZ* vector, Dr. P. Jat for Zip-NeoSVU19tsA58, Drs. A. Kralli and P. Tontonoz for discussions, and A. Papas, Dr. E. Dominguez, and M. Chedwell for technical support.

## Supporting Information

Figure S1 **Schematic of the PPARγ CS1-5_** ***LacZ*** **reporter transgene.** Mouse PPARγ conserved elements 1 to 5 (CS1 to CS5, white boxes) shown in their respective genomic positions (PPARγ exons are numbered and shown in black) were cloned by PCR into a vector containing a minimal Hsp68 promoter upstream of the *LacZ* gene. The transgene shown in the right was excised from this vector and microinjected into C57BL/6 single-cell embryos to generate multiple lines of PPARγ CS1-5_*LacZ* reporter mice.
(TIF)

Figure S2 **Enlarged views of X-gal stained PPARγ (+/−) and PPARγ CS1-5_** ***LacZ*** **transgenic embryos at E14.5.** Note that the stain in line 1 extends beyond the BAT depot to what appear to be capillaries in the dermis. A similar, but weaker, vasculature-like stain is also evident in line 7 embryos.
(TIF)

Figure S3 **Conserved PPARγ sequences 1 to 5 are preferentially active in the stromal-vascular fraction of transgenic line 7.** Real-time qPCR analysis of *LacZ,* PPARγ, and adiponectin expression in the stroma-vascular (SVF) and adipocyte fractions of fat pads derived from PPARγ CS1-5_*LacZ* line 7 mice (6 weeks, n = 3). Error bars denote mean ± S.D.
(TIF)

Figure S4 **Conditionally immortalized SVF cells from PPARγ CS1-5_** ***LacZ*** **transgenic mice differentiate normally into adipocytes.** (*A*) Western blot analysis to determine the time course of T Antigen degradation upon transfer of confluent SVF cells derived from transgenic WAT and BAT depots from the permissive (33°C) to the non-permissive temperature (37°C). (*B*) Phase contrast and Nile red images of cells at day 8 after the induction of adipocyte differentiation shows that cells derived from PPARγ CS1-5_*LacZ* transgenic adipose depots differentiate normally into adipocytes.
(TIF)

Figure S5 **Conserved** **PPARγ elements CS1 to 5 are not transcriptionally active in the vasculature of non-adipose tissues.** Sections of liver, skeletal muscle, and spleen of PPARγ CS1-5_LacZ line 1 transgenics (two sections per tissue) that were X-gal stained upon tissue harvest. Note that no blue cells are evident, indicating that the PPARγ CS1-5 elements do not drive *LacZ* expression in these tissues. Arrows point to some examples of vessels found within the sections.
(TIF)

Table S1 **Genomic location of conserved PPARγ sequences 1 to 5.** Coordinates gathered from the UCSC server, mouse genome released in July 2007. Complete sequences may be downloaded from the server using the coordinates shown.
(DOC)

Methods S1 **Supplemental Methods.**
(DOC)
