# Supplementary figures and images for: Identification of Regulatory Elements That Control PPARγ Expression in Adipocyte Progenitors
Source: PLoS One. 2013 Aug 29;8(8):e72511. doi: 10.1371/journal.pone.0072511 (PMC3757023; doi:10.1371/journal.pone.0072511)

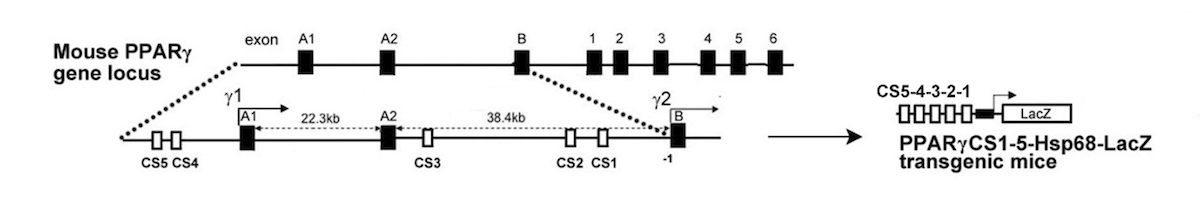

Supplement: Figure S1 — Schematic of the PPARγ CS1-5_ LacZ reporter transgene. Mouse PPARγ conserved elements 1 to 5 (CS1 to CS5, white boxes) shown in their respective genomic positions (PPARγ exons are numbered and shown in black) were cloned by PCR into a vector containing a minimal Hsp68 promoter upstream of the LacZ gene. The transgene shown in the right was excised from this vector and microinjected into C57BL/6 single-cell embryos to generate multiple lines of PPARγ CS1-5_LacZ reporter mice. (TIF) [file pone.0072511.s001.tif]

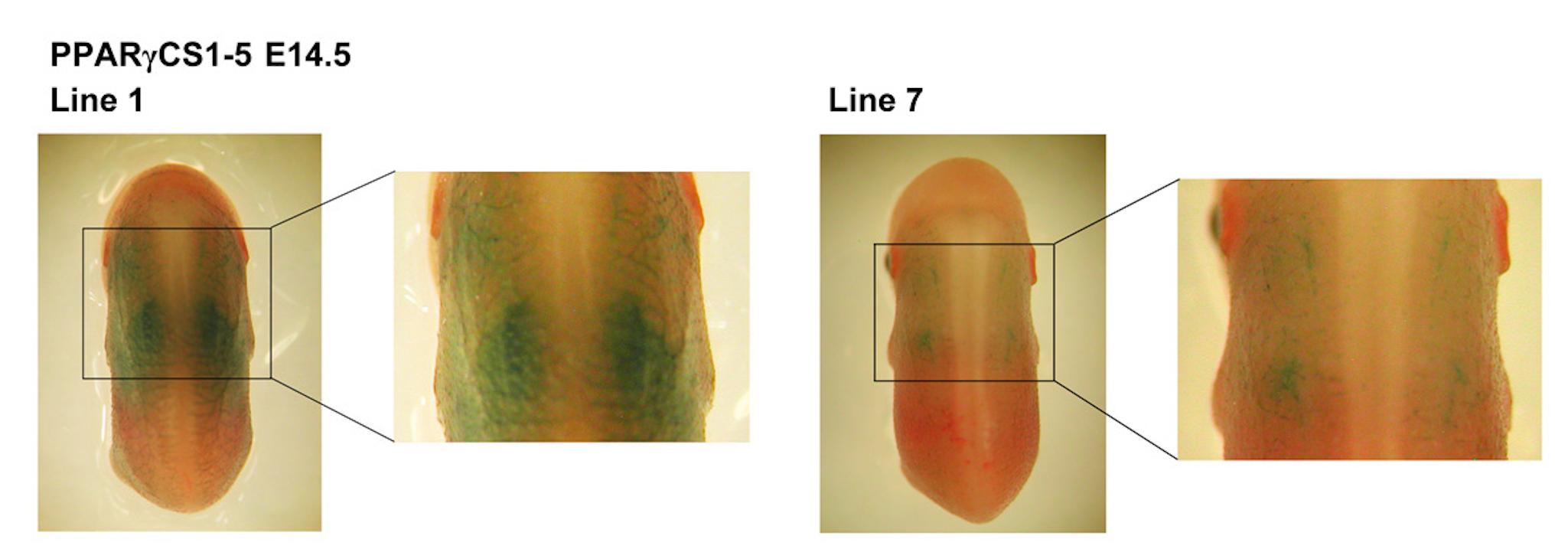

Supplement: Figure S2 — Enlarged views of X-gal stained PPARγ (+/−) and PPARγ CS1-5_ LacZ transgenic embryos at E14.5. Note that the stain in line 1 extends beyond the BAT depot to what appear to be capillaries in the dermis. A similar, but weaker, vasculature-like stain is also evident in line 7 embryos. (TIF) [file pone.0072511.s002.tif]

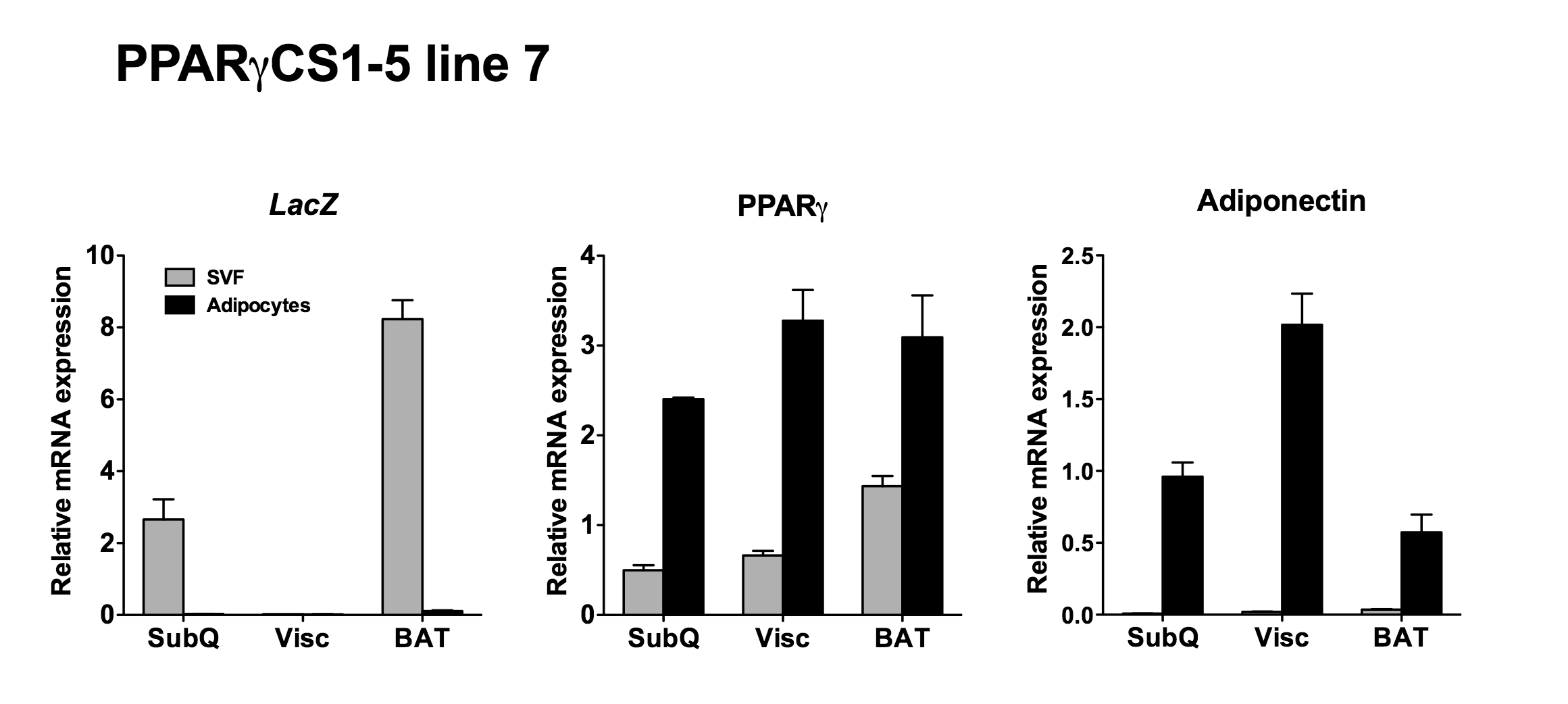

Supplement: Figure S3 — Conserved PPARγ sequences 1 to 5 are preferentially active in the stromal-vascular fraction of transgenic line 7. Real-time qPCR analysis of LacZ, PPARγ, and adiponectin expression in the stroma-vascular (SVF) and adipocyte fractions of fat pads derived from PPARγ CS1-5_LacZ line 7 mice (6 weeks, n = 3). Error bars denote mean ± S.D. (TIF) [file pone.0072511.s003.tif]

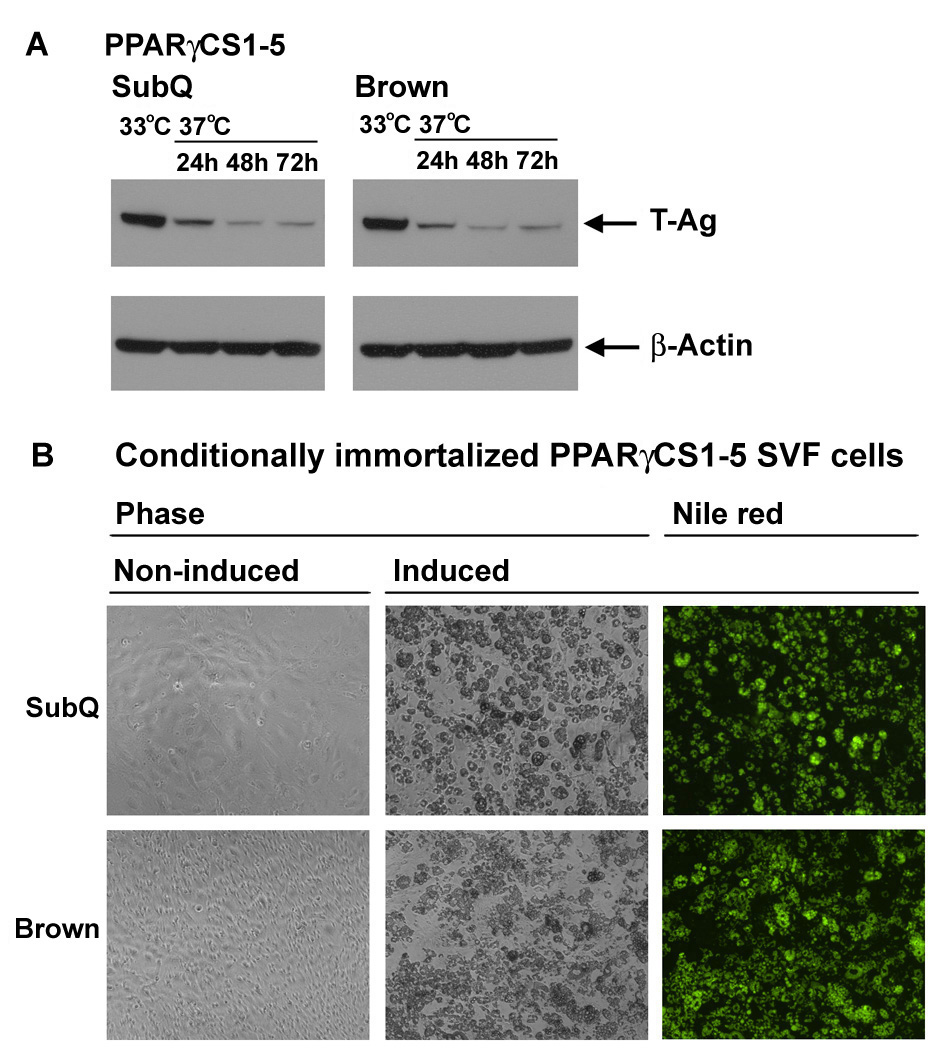

Supplement: Figure S4 — Conditionally immortalized SVF cells from PPARγ CS1-5_ LacZ transgenic mice differentiate normally into adipocytes. (A) Western blot analysis to determine the time course of T Antigen degradation upon transfer of confluent SVF cells derived from transgenic WAT and BAT depots from the permissive (33°C) to the non-permissive temperature (37°C). (B) Phase contrast and Nile red images of cells at day 8 after the induction of adipocyte differentiation shows that cells derived from PPARγ CS1-5_LacZ transgenic adipose depots differentiate normally into adipocytes. (TIF) [file pone.0072511.s004.tif]

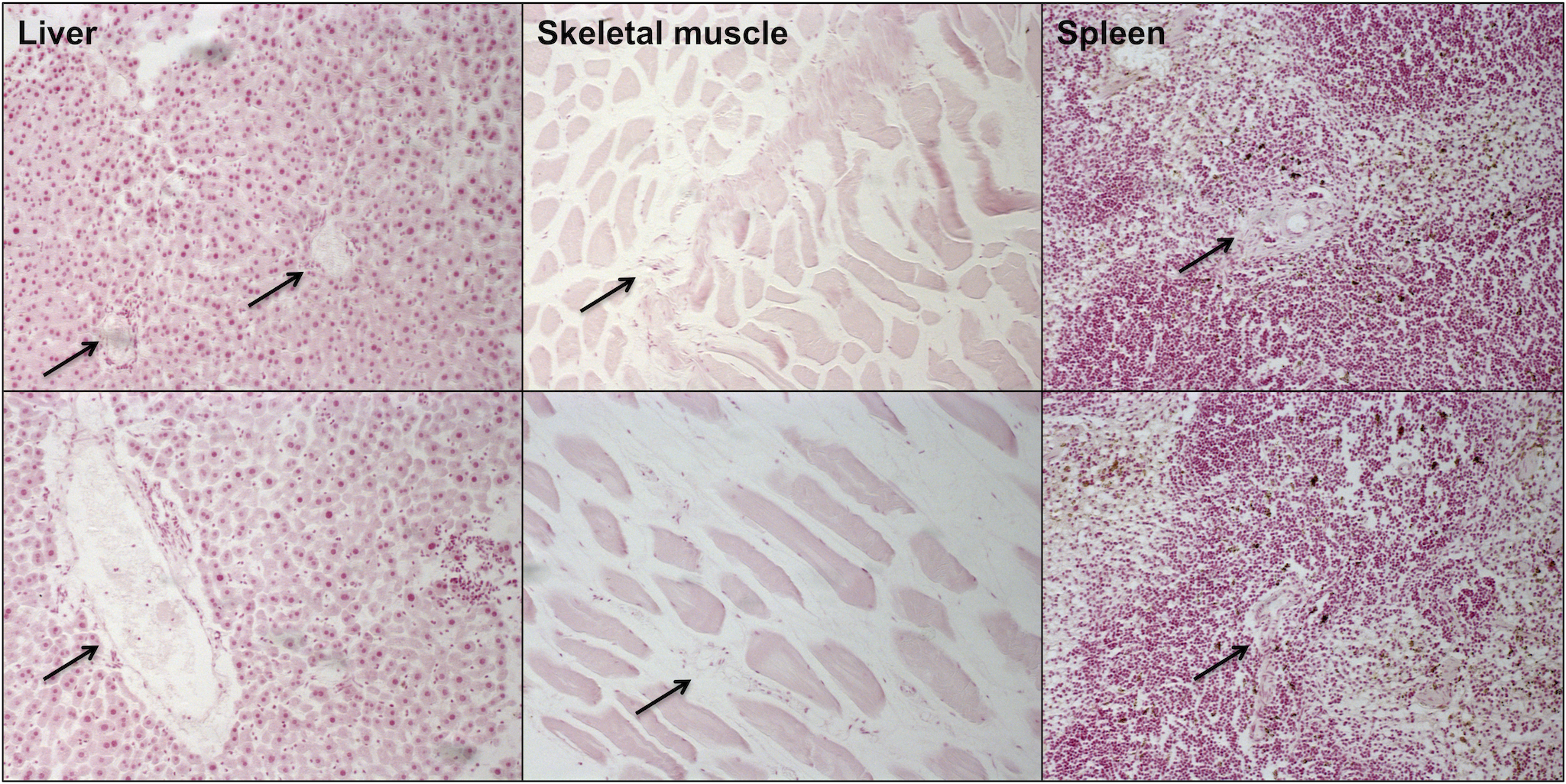

Supplement: Figure S5 — Conserved PPARγ elements CS1 to 5 are not transcriptionally active in the vasculature of non-adipose tissues. Sections of liver, skeletal muscle, and spleen of PPARγ CS1-5_LacZ line 1 transgenics (two sections per tissue) that were X-gal stained upon tissue harvest. Note that no blue cells are evident, indicating that the PPARγ CS1-5 elements do not drive LacZ expression in these tissues. Arrows point to some examples of vessels found within the sections. (TIF) [file pone.0072511.s005.tif]
