# Supplementary material for: Identification of Regulatory Elements That Control PPARγ Expression in Adipocyte Progenitors
Source: PLoS One. 2013 Aug 29;8(8):e72511. doi: 10.1371/journal.pone.0072511 (PMC3757023; doi:10.1371/journal.pone.0072511)
Supplement: Table S1 — Genomic location of conserved PPARγ sequences 1 to 5. Coordinates gathered from the UCSC server, mouse genome released in July 2007. Complete sequences may be downloaded from the server using the coordinates shown. (DOC) [file pone.0072511.s006.doc]

**Table S1**

**Genomic location of conserved PPARγ sequences 1 to 5**

| **NCBI37/mm9** | **distance from γ2 TSS** | **chromosome** | **start** | **end** | **length** |
| --- | --- | --- | --- | --- | --- |
| CS1 | -11 kb | 6 | 115361229 | 115362078 | 849 bp |
| CS2 | -15 kb | 6 | 115356189 | 115356900 | 711 bp |
| CS3 | -32 kb | 6 | 115339651 | 115340642 | 991 bp |
| CS4 | -76 kb | 6 | 115295740 | 115296097 | 357 bp |
| CS5 | -79 kb | 6 | 115292170 | 115292623 | 453 bp |

**Supplementary Methods**

***Conditional immortalization of SV cells and induction of adipocyte differentiation*.** Isolated SV cells from subcutaneous and brown adipose tissue of transgenic and control mice were infected with a retrovirus encoding a temperature sensitive SV40 large T Antigen [[1](#_ENREF_1),[2](#_ENREF_2)]. A retroviral SV40 T Ag expression construct (VGIP3-tsA58) was generated by subcloning U19tsA58 from Zip-NeoSVU19tsA58 into the VGIP3-Puromycin vector. Retroviruses were packaged by transient transfection in 293T cells. Infected SV cells were selected with puromycin (0.8 μg/mL) at 33°C (permissive temperature) for 7-10 days to immortalize them. Immortalized cells were maintained at 33°C. For adipogenesis assays, cells were transferred to 37°C (non-permissive temperature) 2 days prior to confluence to allow degradation of T Antigen. White adipogenesis was induced in cells cultured in DMEM/F12 10% FBS medium by treatment with 1 μM dexamethasone, 0.5 mM IBMX, and 5 μg/mL insulin for three days after confluence. Subsequently, cells were maintained in media plus insulin alone until fully differentiated. To induce differentiation of brown SV immortalized cells, they were cultured in DMEM/F12 medium with 10% FBS supplemented with 20 nM insulin and 1 nM T3 (differentiation medium) for 2 days, and then treated for 2 days with differentiation medium further supplemented with 0.5 mM IBMX, 0.5 μM dexamethasone, and 0.125 mM indomethacin. After induction, cells were maintained in differentiation medium.

***Histology*.** Excised adipose tissues were post-fixed in Zn-formalin overnight, dehydrated, embedded in paraffin, and sectioned with a microtome at 10 μm. Nuclear fast red was used for counterstaining of paraffin-embedded sections. Images were obtained using Leica SCN400 Digital Slide Scanner.

**References**

1. Morganstein DL, Christian M, Turner JJ, Parker MG, White R (2008) Conditionally immortalized white preadipocytes: a novel adipocyte model. J Lipid Res 49: 679-685.

2. Jat PS, Noble MD, Ataliotis P, Tanaka Y, Yannoutsos N, et al. (1991) Direct derivation of conditionally immortal cell lines from an H-2Kb-tsA58 transgenic mouse. Proc Natl Acad Sci U S A 88: 5096-5100.
